# A Genome-Wide Genetic Diversity Scan Reveals Multiple Signatures of Selection in a European Soybean Collection Compared to Chinese Collections of Wild and Cultivated Soybean Accessions

**DOI:** 10.3389/fpls.2021.631767

**Published:** 2021-02-26

**Authors:** Aamir Saleem, Hilde Muylle, Jonas Aper, Tom Ruttink, Jiao Wang, Deyue Yu, Isabel Roldán-Ruiz

**Affiliations:** ^1^Plant Sciences Unit, Flanders Research Institute for Agriculture, Fisheries and Food (ILVO), Melle, Belgium; ^2^Department of Plant Biotechnology and Bioinformatics, Ghent University, Ghent, Belgium; ^3^National Center for Soybean Improvement, National Key Laboratory of Crop Genetics and Germplasm Enhancement, Nanjing Agricultural University, Nanjing, China

**Keywords:** selective sweeps, breeding, European soybean, genetic diversity, Chinese soybean, *Glycine max*, SNP markers, signatures of selection

## Abstract

Targeted and untargeted selections including domestication and breeding efforts can reduce genetic diversity in breeding germplasm and create selective sweeps in crop genomes. The genomic regions at which selective sweeps are detected can reveal important information about signatures of selection. We have analyzed the genetic diversity within a soybean germplasm collection relevant for breeding in Europe (the EUCLEG collection), and have identified selective sweeps through a genome-wide scan comparing that collection to Chinese soybean collections. This work involved genotyping of 480 EUCLEG soybean accessions, including 210 improved varieties, 216 breeding lines and 54 landraces using the 355K SoySNP microarray. SNP calling of 477 EUCLEG accessions together with 328 Chinese soybean accessions identified 224,993 high-quality SNP markers. Population structure analysis revealed a clear differentiation between the EUCLEG collection and the Chinese materials. Further, the EUCLEG collection was sub-structured into five subgroups that were differentiated by geographical origin. No clear association between subgroups and maturity group was detected. The genetic diversity was lower in the EUCLEG collection compared to the Chinese collections. Selective sweep analysis revealed 23 selective sweep regions distributed over 12 chromosomes. Co-localization of these selective sweep regions with previously reported QTLs and genes revealed that various signatures of selection in the EUCLEG collection may be related to domestication and improvement traits including seed protein and oil content, phenology, nitrogen fixation, yield components, diseases resistance and quality. No signatures of selection related to stem determinacy were detected. In addition, absence of signatures of selection for a substantial number of QTLs related to yield, protein content, oil content and phenological traits suggests the presence of substantial genetic diversity in the EUCLEG collection. Taken together, the results obtained demonstrate that the available genetic diversity in the EUCLEG collection can be further exploited for research and breeding purposes. However, incorporation of exotic material can be considered to broaden its genetic base.

## Introduction

Crop improvement relies on genetic diversity of plant genetic resources. A high genetic diversity provides an opportunity for plant breeders to develop cultivars with desirable characteristics (Savchenko, 2017; Byrne et al., 2018). Today’s improved cultivars of various crops, with specific characteristics depending on their use and environmental adaptation, are the result of historical domestication events and intentional as well as unintentional selections by farmers and breeders (Bradshaw, 2017; Stoskopf et al., 2019). The processes of domestication and selection lead to an increased frequency of favorable alleles, and in the most extreme situation may cause complete fixation at genomic loci underlying beneficial traits in the genepool of the crop (Smýkal et al., 2018; Weigand and Leese, 2018). At chromosomal scale, the locus that underlies a beneficial trait is surrounded by other linked loci carrying neutral mutations. The selection process targeting the advantageous allele also results in an increase of the frequency of alleles at those neutral loci that are in linkage disequilibrium with the advantageous allele. This causes a so-called “selective sweep” in the genome. Genomic regions that have undergone a selective sweep are characterized by high levels of homozygosity, an increase in low- and high-frequency alleles, a high linkage disequilibrium and a low genetic diversity (Nielsen, 2005; Hufford et al., 2012).

Selective sweeps have been analyzed in several crops to reconstruct their history of domestication and selection and to identify the genetic loci and their underlying genome sequence that were mainly affected by these processes. For example, Hufford et al. (2012) identified 3,040 genes through selective sweep analysis, revealing signatures of domestication and improvement of maize in the United States. The dispersion history and adaptive evolution of wheat throughout the agro-ecological zones of China have been inferred by population genetics analysis including selective sweep analysis (Zhou et al., 2018). Ndjiondjop et al. (2019) identified 37 candidate selective sweep regions harboring genes related to biotic and abiotic stress tolerance in African rice, and demonstrated that those regions displayed low genetic diversity as a result of strong positive selection and domestication in African rice compared to Asian rice and its wild progenitor (Oryza barthii A. Chev.). A selective sweep analysis in barley by Pankin et al. (2018) provided molecular evidence of multiple domestication origins and allowed to distinguish domestication-related traits (i.e., non-brittle rachis) from improvement-related traits (i.e., naked grain).

Modern cultivated soybean was domesticated approximately 5000 years ago from its wild progenitor Glycine soja, which is distributed throughout Eastern Asia, including most of China, South Korea, and Japan (Jeong et al., 2019). Soybean is the world’s most grown high-value legume crop with beans containing high percentages of protein and oil (Pagano and Miransari, 2016). Being a restorative crop, soybean fixes atmospheric nitrogen in symbiosis with Rhizobium bacteria and delivers environmental services by minimizing the need for mineral nitrogen fertilizer. Between 2008 and 2018, global soybean production has grown from 212 to 337 million tons per year, while the total cultivated surface increased from 97 to 124 million hectares (IDH and IUCN NL, 2019). Soybean was first introduced to Europe during the second half of the 19th century. The current soybean acreage of 5.5 million hectares in Europe, representing a mere 3.4% of the world soybean production (FAOSTAT., 2019), can meet only 34% of the current European need for soybean (IDH and IUCN NL, 2019)). To meet the increasing European demand and to reduce the dependency on import, it is crucial to expand soybean cultivation and to adapt soybean genotypes to new cultivation zones in Europe. This requires a good understanding of the origin and genetic architecture of European soybean germplasm and how it relates to the germplasm from other origins.

Based on the responsiveness of soybean flowering and maturity to photoperiod and temperature, a total of 13 distinct maturity groups have been defined, of which only early maturing types (maturity groups MG000 to MGII) are suitable for cultivation in Europe (Kurasch et al., 2017). Previous studies have shown a narrow genetic base of the European soybean germplasm (Hahn and Würschum, 2014; Žulj Mihaljević et al., 2020), which can be due to the use of only a few ancestors originating from Canada, North America, Japan and China for breeding in Europe (Ristova et al., 2010; Hahn and Würschum, 2014; Miladinović et al., 2018). In addition, the original material used for breeding probably carried a low level of genetic diversity, as both pedigree and molecular marker data have indicated a narrow genetic base of North American and Canadian germplasm (Gizlice et al., 1996; Vaughn and Li, 2016; Bruce et al., 2019). In contrast, the Chinese soybean breeding pool contains a high level of genetic diversity because of a long history of cultivation over diverse eco-geographical zones with varying ranges of temperature and photoperiod (Liu et al., 2017). Selective sweep analysis has also been applied in soybean to understand the domestication and selection history. For example, Wen et al. (2015); Zhou et al. (2015) and Wang et al. (2016) report candidate selection regions harboring genes potentially involved in traits of agronomic relevance such as grain yield, seed size, flowering date, maturity date, seed protein and oil content and traits related to stress tolerance. In addition, selective sweep analysis by Jeong et al. (2019) reported domestication-related signals in soybean using mainly germplasm from Japan and Korea. Thus, available studies have considered materials of Chinese, Japanese and Korean origin. However, similar studies have not been performed in the soybean genepool available in Europe.

Our current knowledge on the origin and genetic relationships within European soybean germplasm is still fragmented. Main reasons are the limited number of accessions (covering only a fraction of the total genetic diversity) that were included in previous studies (e.g., 28 accessions in Ristova et al. (2010), 93 in Hahn and Würschum (2014), 75 in Kurasch et al. (2017) and 97 in Žulj Mihaljević et al. (2020)), and/or the low number of genetic markers that were used for screening. Miladinović et al. (2018), genotyped 445 accessions at 85,000 SNP loci, but only used the 38 SNPs located in maturity genes for analysis, and focused on materials from one European breeding program. To fill this gap in our knowledge about the genetics of soybean germplasm relevant for breeding in Europe and to develop breeding tools for legume crops including soybean, a consortium was established within the European Union project EUCLEG^1^. In this context a unique collection of 480 soybean accessions considered relevant for European breeders, originating from 25 countries and covering a broad range of genetic diversity was assembled (named the EUCLEG collection in what follows). This offers a unique opportunity to compare the genetic diversity of the EUCLEG collection to that contained in reference materials from China, helping us to understand the main forces that have shaped the soybean genepool currently being used in breeding programs outside China.

Here, we present an analysis of the genetic diversity within the EUCLEG collection and identify selective sweeps through a genome-wide diversity scan between the EUCLEG and a Chinese soybean collection (Wang et al., 2016) (“NJAU collection”). Specific objectives of this study were: (i) to explore the structure and genetic relatedness of accessions in the EUCLEG collection; (ii) to determine the level of genetic diversity in the EUCLEG collection compared to that of the NJAU collection; (ii) to identify genomic regions that putatively underwent selective sweeps in the EUCLEG collection and their significance for future soybean improvement efforts in Europe.

## Materials and Methods

### EUCLEG Collection

The EUCLEG collection consists of 480 accessions belonging to maturity groups (MG) 000, 00, 0 and I/II, and includes 210 improved varieties, 216 breeding lines and 54 landraces. A detailed description of this collection is provided in Supplementary Table 1. This collection was made in collaboration with seed companies and gene banks by considering the accessions with diverse geographical origins, with varying levels of selection (landraces, varieties and breeding lines), and their relevance for European soybean breeders.

For DNA extraction, one fully developed unifoliate leaf was sampled per accession from plants grown in the field. Leaf samples were lyophilized and stored under vacuum conditions until use. Genomic DNA was extracted using the DNeasy^®^ Plant Mini Kit^2^. Pure and good quality DNA samples with an average concentration of 106 ng/μl (range 24–731 ng/μl) were used for genotyping using the 355K SoySNP Axiom microarray (Wang et al., 2016) from Affymetrix (Thermo Fisher Scientific), via Eurofins, DK.

### NJAU Collection

The NJAU collection originates from the Germplasm Storage of Chinese National Center for Soybean Improvement, Nanjing Agricultural University, China, and comprises 122 wild and 272 cultivated accessions. It covers the three ecological habitats of soybean in China including the regions of Northern China, Huang-Huai and Southern China. A full description of the NJAU collection is available in Wang et al. (2016). This collection has previously been genotyped using the 355K SoySNP microarray (Wang et al., 2016). Here, we have combined the raw microarray fluorescence data of the EUCLEG samples with the raw fluorescence data of the NJAU samples to perform a “joint” SNP calling.

### SNP Calling

SNP calling was performed using the software Axiom Analysis Suite (AAS) from Affymetrix^®^, following the instructions provided in the Axiom Analysis Suite 3.1 user guide^3^. Before SNP calling of the 874 samples of the combined data set (EUCLEG and NJAU), we first checked the performance of the 355K SoySNP microarray on the 480 EUCLEG samples separately. This step was considered necessary, as the 355K SoySNP microarray was developed using the NJAU collection and might perform sub-optimally with plant materials of a different origin. In brief, the Affymetrix^®^ Power Tools (APT) software package, version 1.15.0 implemented in AAS performed sample quality control based on 20,000 non-polymorphic probe sets and considering the parameters Dish Quality Control (DQC; determines the intensity of contrast between signal and noise) and Sample Call Rate (QC-CR; refers to the ratio of genotype-called SNPs to attempted SNPs in a sample). Based on criteria DQC > 0.82 and QC-CR ≥ 97, AAS filtered out four poor-quality samples. The R package SNPolisher version 1.3.6.7 implemented in AAS was used for SNP calling using 609,883 probe sets targeting 355,595 SNPs. Its Ps_Classification function classified the SNPs/probe sets into six categories based on the following SNP QC metrics: call rate (CR) ≥97%, Fisher’s linear discriminant (FLD) ≥ 3.6, heterozygous strength offset (HetSO) ≥−0.1, and homozygote ratio offset (HomRO) ≥0.3 for one-cluster or two-cluster SNPs or ≥−0.9 for three-cluster SNPs. A summary of the SNP classification was obtained for the 476 good quality samples of the EUCLEG collection. We compared this summary with the SNP classification summary obtained from the NJAU collection by Wang et al. (2016).

In a second step, we genotyped the combined dataset (EUCLEG and NJAU), starting from the raw fluorescence data following the procedure described above. In the quality control step, 69 poor quality samples were excluded. SNP calling was performed on the remaining 805 good quality samples. After genotyping, low quality SNPs based on SNP QC metrics were excluded and a final genotyping dataset containing 229,557 SNPs was generated. This dataset was divided in three subsets for further processing: EUCLEG, NJAU-Wild and NJAU-Cultivated, comprising 477, 82, and 246 accessions, respectively. For the divisions NJAU-Wild and NJAU-Cultivated, we refer to Wang et al. (2016). In further analyses, we considered either the whole collection (EUCLEG and NJAU) or some of these subsets.

For some of the downstream analyses, the genomic coordinates of the SNPs were required. Because during the development of the 355K SoySNP microarray SNP coordinates were assigned using an older version of the soybean reference genome sequence (Glyma.Wm82.a1), we positioned SNPs onto the novel reference genome sequence Glyma.Wm82.a2 (with improved assembly and gene annotation quality compared to Glyma.Wm82.a1). Finally, the 224,993 SNPs corresponding to probes that could be positioned onto the 20 soybean chromosomes using a blast query were considered for further analyses.

### Population Structure Analysis

The results of population structure of the NJAU collection are available in Wang et al. (2016). Here, we performed a population structure analysis of EUCLEG and NJAU combined (805 accessions in total). Two approaches were applied. In the first approach, a model-based structure analysis was performed in fastSTRUCTURE 1.0 (Raj et al., 2014) including 179,812 SNPs with minor allele frequency (MAF) of at least 5% across the sample set. The K value was varied from 2 to 10, while for other parameters default settings were used. The optimum value of K was determined using the best marginal likelihood value of fastSTRUCTURE-output from K = 2 to K = 10. The results of fastSTRUCTURE were graphically visualized using the R-package pophelper v. 2.1.0 (Francis, 2017). In the second approach, a principal component analysis (PCA) was performed in TASSEL 5 (Bradbury et al., 2007). The missing values of SNPs were imputed using the unweighted average method and PCA was performed on the genetic correlation matrix of accessions considering the first five principal components.

Population structure was also inferred for the 477 genotyped accessions of the EUCLEG collection separately, using the settings and methods described in the previous paragraph. The analysis included 139,986 SNPs with MAF of at least 5% across the sample set. The number of subgroups (K) was determined considering the delta log-likelihood criterion. For the interpretation of the results of fastSTRUCTURE, once an optimal value of K was identified, each accession was assigned to a subgroup “n” for which the ancestral coefficient reached a value Qn ≥ 0.4; where n is the number of subgroup (1 to K). Accessions for which the two highest Q values differed by less than 0.2 were considered “Admixed”. Finally, the degree of divergence between the EUCLEG and the NJAU collections was estimated by calculating a fixation index (F_*ST*_) value per SNP site in VCFtools v. 0.1.15 (Danecek et al., 2011) following the methods of Weir and Cockerham (1984).

Hierarchical cluster analysis of EUCLEG and NJAU combined was performed to determine the relationship among accessions of both collections, and to see the relationship between the EUCLEG part from the Chinese origin with other accessions. For this, a dendrogram was constructed following Ward’s D2 method (Murtagh and Legendre, 2014) and using Nei’s standard genetic distances between accessions (Nei, 1972).

### Genetic Diversity Estimates

Linkage disequilibrium (LD) analysis was performed in VCFtools v. 0.1.15 considering the filtered genotyping data including 139,986 SNPs, 162,098 SNPs and 185,194 SNPs with MAF of at least 5% in the sample sets EUCLEG, NJAU-Cultivated, and NJAU-Wild, respectively. LD was estimated for each chromosome by computing the *r*^2^ for all pairwise comparisons of two SNPs located at a maximum of 1000 kbp inter-SNP distance. The LD decay distance per chromosome was estimated as the point at which *r*^2^ dropped to half of its maximum value. The genome-wide LD decay was estimated by pooling the LD output across all chromosomes.

Diversity estimates were determined for EUCLEG, NJAU-Cultivated, and NJAU-Wild separately, including all 224,993 SNPs. The EUCLEG collection contained 21 accessions from Chinese origin, but to avoid any possible confounding effect of those accessions while comparing genetic diversity between EUCLEG and NJAU, these 21 accessions were not considered. The average pairwise divergence among genotypes within each collection was then determined by computing the nucleotide diversity index (π) per SNP site in VCFtools v. 0.1.15.

### Selective Sweep Analysis

To detect signals of selection in the EUCLEG collection, the cross-population composite likelihood ratio test (XP-CLR) implemented in XP-CLR v. 1.0 (Chen et al., 2010) was used. XP-CLR is a site frequency spectrum (SFS)-based method that detects selective sweeps by jointly modeling the multi-locus allele frequency differentiation between two populations. Given the allele frequency of a locus in the reference population, XP-CLR maximizes the likelihood ratio of the allele frequency in an object population between a selective sweep model and a null model (Chen et al., 2010). We compared the object EUCLEG collection to the reference NJAU-Wild collection. The 21 accessions of Chinese origin in the EUCLEG collection were not considered in this analysis. XP-CLR requires as input the genetic position (expressed in recombination units) of each SNP. Because for most SNPs the genetic position was unknown, we transformed physical positions (Mbp) to recombination positions (cM) considering a homogeneous recombination rate (1 Mbp = 1 cM) throughout the soybean genome.

The whole genome was scanned in XP-CLR choosing a sliding window of 1 Mbp at steps of 5 kbp. XP-CLR options were as follows: XPCLR -xpclr genofile1.txt genofile2.txt mapfile outputfile -w1 snpWin 0.01 gridSize 5000 chrN -p0 corrLevel 0.95; where genofile1.txt and genofile2.txt correspond to the object (EUCLEG) and reference (NJAU-Wild) collections, respectively. Because two SNP loci with high pairwise *r*^2^ values can provide redundant information, corrLevel was set to 0.95 to weight the XP-CLR value of a window containing highly correlated SNPs. Windows with weighted XP-CLR scores in the top 1% of the empirical distribution of the genome-wide XP-CLR values were used to delineate regions of interest. To define the regions of interest (hereafter called selective sweep regions), we combined neighboring windows when the gap was less than 1 Mbp.

SoyBase^4^ (Grant et al., 2010) was used to search for reported QTLs located in the selective sweep regions and to generate a list of genes positioned in respective selective sweep regions. The transcripts description was obtained from “Gmax_275_Wm82.a2.v1.annotation_info.txt”^5^ and the annotation of biological functions was obtained from UniProtKB^6^. The former contains transcript definition of the best hit obtained through homology-dependent sequence analysis of soybean transcripts in the Arabidopsis genome, whereas the latter corresponds to their molecular and biological functions manually annotated and reviewed from literature and computational analysis by the UniProtKB.

## Results

### Evaluation of the Use of the 355K SoySNP Array in the EUCLEG Collection

Analysis of the EUCLEG collection with the 355K SoySNP array revealed a total of 285,953 SNP markers (80% of the total 355,595 SNPs present on the array) belonging to the recommended categories including PolyHighResolution (PHR, total 211,593), MonoHighResolution (MHR, total 46,953) and NoMinorHom (NMH, total 27,407). These categories refer to SNPs exhibiting all three genotypic classes with a good cluster resolution (PHR), SNPs with good cluster resolution but displaying only one of the homozygous clusters (MHR) and SNPs with good cluster resolution but for which one of the two homozygous clusters is missing (NMH). These proportions correspond quite well with those previously reported for the NJAU collection by Wang et al. (2016) (Supplementary Figure 1), indicating that the 355K SoySNP array is not only useful for the genotyping of Chinese soybean germplasm, but also for germplasm from other origins.

Genotyping of the EUCLEG and NJAU combined collection rendered 229,557 SNPs (65% of the total 355,595) belonging to the recommended categories (PHR, MHR and NMH; a total of 194,171, 16,868 and 18,518 respectively). For the remaining 126,038 SNPs, at least one of the QC metrics were below the threshold and hence they were assigned to non-recommended categories. There were 16% (of the total 355,595) more SNPs of non-recommended categories in the combined analysis as compared to the separate analysis of the EUCLEG collection. This was because a number of SNPs (19% of the total 355,595) of recommended categories (PHR, MHR and NMH) in the EUCLEG separate analysis were assigned to the non-recommended categories in the combined analysis. Taken together, these results indicate that SNP calling on EUCLEG and NJAU combined is essential to get a more precise classification of SNP markers as compared to a separate analysis for each collection. In addition, these results indicate that the SNP dataset of EUCLEG and NJAU combined contains a high number of SNPs from the recommended categories.

For 224,993 SNPs of the total 229,557 (98%), new coordinates could be positioned onto 20 chromosomes in the Glyma.Wm82.a2 soybean reference genome sequence using a BLAST query. The probes targeting the remaining SNPs (4,564) were either missing in the novel soybean genome assembly or were assigned to the non-anchored scaffolds and not to chromosomes, and were excluded from subsequent analyses. The genome-wide distribution of the final set of 224,993 SNPs used for downstream data analyses is given in Table 1. The longest chromosome (18) contained the highest number of SNPs (6.3% of the total 224,993), and the shortest chromosome (11) contained the lowest number of SNPs (3.8% of the total 224,993). The average SNP density was the lowest on chromosome 1 and the highest on chromosome 13 (19 and 27 SNPs per 100 kbp, respectively). The average distance between two adjacent SNPs was 2.6 kbp (Table 1 and Supplementary Figure 2).

**TABLE 1:** Genomic distribution of the 224,993 SNPs considered in this study and their distribution across the 20 chromosomes of Glycine max.

**Chromosome**	**Length (bp)**	**Number of SNPs**	**SNP Density^*a*^**	**SNP Spacing^*b*^ (bp)**	**Polymorphic SNPs^*c*^**			**LD decay distance (kbp)**			**π**		
					**EUCLEG**	**NJAU-Cultivated**	**NJAU-Wild**	**EUCLEG**	**NJAU-Cultivated**	**NJAU-Wild**	**EUCLEG**	**NJAU-Cultivated**	**NJAU-Wild**
1	56,831,624	11,255	19(54)	3,099	0.77	0.86	0.89	175	145	75	0.20	0.26	0.31
2	48,577,505	11,994	24(57)	2,626	0.85	0.88	0.90	165	105	35	0.28	0.28	0.30
3	45,779,781	10,711	23(57)	2,548	0.79	0.85	0.91	145	80	45	0.24	0.26	0.31
4	52,389,146	10,782	20(56)	2,946	0.83	0.87	0.88	190	100	45	0.29	0.25	0.29
5	42,234,498	9,938	23(72)	2,647	0.73	0.86	0.91	160	100	50	0.20	0.25	0.34
6	51,416,486	12,176	23(60)	2,620	0.82	0.87	0.87	175	75	35	0.24	0.25	0.28
7	44,630,646	10,618	23(88)	2,666	0.80	0.85	0.90	165	90	40	0.22	0.27	0.31
8	47,837,940	12,145	25(71)	2,482	0.79	0.88	0.92	160	80	30	0.20	0.26	0.32
9	50,189,764	11,901	23(59)	2,666	0.84	0.85	0.90	170	105	40	0.27	0.29	0.31
10	51,566,898	11,717	22(67)	2,840	0.80	0.86	0.89	135	105	85	0.23	0.26	0.32
11	34,766,867	8,536	24(68)	2,512	0.74	0.86	0.91	110	60	50	0.17	0.24	0.31
12	40,091,314	9,846	24(63)	2,467	0.68	0.86	0.91	235	95	60	0.19	0.19	0.33
13	45,874,162	12,658	27(80)	2,339	0.78	0.88	0.91	155	75	20	0.24	0.26	0.30
14	49,042,192	11,730	23(63)	2,476	0.81	0.86	0.89	225	100	140	0.23	0.23	0.33
15	51,756,343	11,776	22(57)	2,767	0.85	0.88	0.91	265	120	45	0.26	0.28	0.30
16	37,887,014	9,683	25(62)	2,316	0.86	0.89	0.90	261	115	55	0.27	0.29	0.31
17	41,641,366	10,598	25(71)	2,543	0.84	0.88	0.91	140	100	40	0.24	0.30	0.30
18	58,018,742	14,389	24(59)	2,266	0.86	0.89	0.91	180	155	65	0.20	0.32	0.29
19	50,746,916	11,960	23(64)	2,446	0.83	0.87	0.91	345	170	70	0.25	0.23	0.33
20	47,904,181	10,580	22(64)	2,615	0.77	0.87	0.90	200	105	70	0.21	0.24	0.32
Average	47,459,169	11,250	23(65)	2,594	0.80	0.87	0.90	188	104	55	0.23	0.26	0.31

*The main characteristics of the SNPs per chromosome are shown, together with the LD decay distance and average nucleotide diversity (π) per chromosome.*

*^*a*^Average number of SNPs per window of 100 kbp; the maximum number of SNPs for a window of 100 kbp is shown between brackets.*

*^*b*^Average distance between neighboring SNPs.*

*^*c*^Proportion of polymorphic SNPs out of total SNPs per chromosome.*

### Population Structure Analysis

**FIGURE 1:**
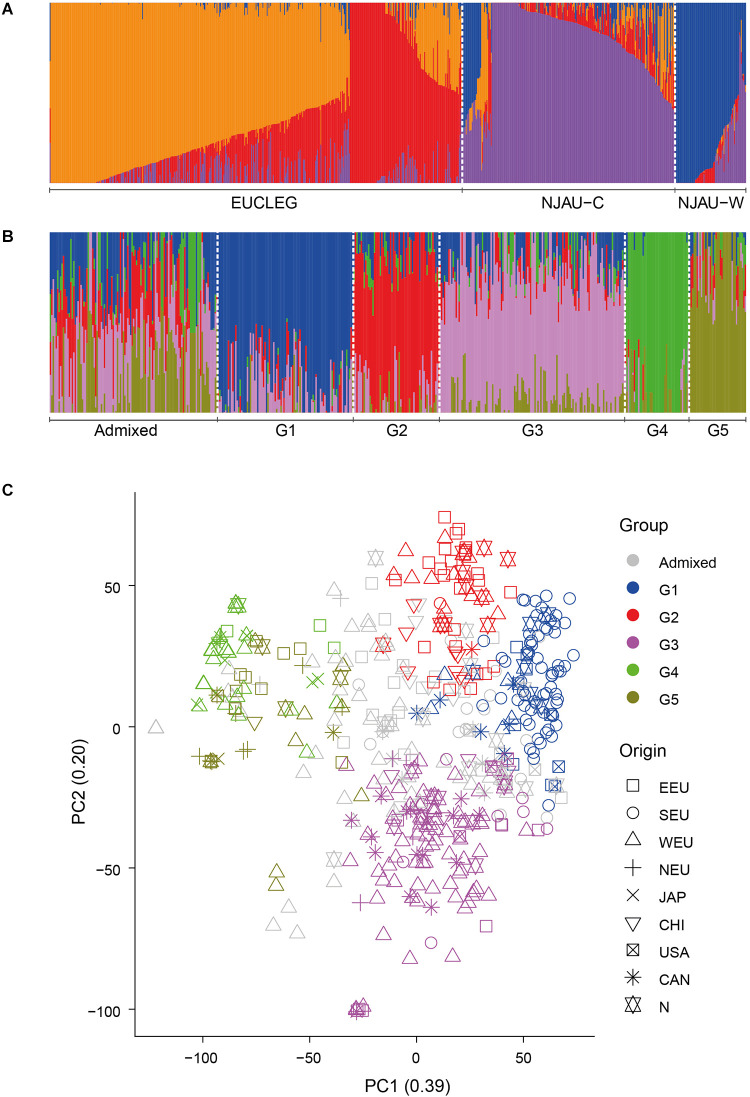
**(A)** Graphical representation of the fastSTRUCTURE results for EUCLEG and NJAU combined sample sets at optimum K = 4. “NJAU-C” and “NJAU-W” represent the NJAU-Cultivated and NJAU-Wild parts of the NJAU collection. **(B)**. Graphical representation of the fastSTRUCTURE results for the EUCLEG collection at optimum K = 5. “G1” to “G5” are the five subgroups identified by fastSTRUCTURE. “Admixed” are the accessions that could not be assigned unequivocally to one of the subgroups (see main text for further details). **(C)**. Graphical representation of the first two dimensions of a principal components analysis for the EUCLEG collection. PC1 and PC2 explained 39 and 20% of total genetic variation in the EUCLEG collection. EEU: Eastern Europe, SEU: Southern Europe, WEU: Western Europe, NEU: Northern Europe, JAP: Japan, CHI: China, USA: United States of America, CAN: Canada, N: Unknown origin.

We analyzed the presence of population structure using two approaches, fastSTRUCTURE and PCA. In the EUCLEG and NJAU combined analysis comprising 805 accessions, the marginal likelihood of the fastSTRUCTURE-output from K = 2 to K = 10 indicated the optimum K between 2 and 4 (Supplementary Figure 3). The EUCLEG sample set clustered separately from NJAU sample sets (Figure 1A and Supplementary Figure 4). The division between NJAU-Cultivated and NJAU-Wild (as defined by Wang et al., 2016) was also apparent. The F_*ST*_ values were 0.14, 0.34 and 0.22 for EUCLEG vs. NJAU-Cultivated, EUCLEG vs. NJAU-Wild and NJAU-Cultivated vs. NJAU-Wild comparisons, respectively. This indicates that the set of 805 soybean accessions considered in this study consists of three major groups including EUCLEG, NJAU-Cultivated and NJAU-Wild. The relatively low F_ST_ between EUCLEG and NJAU-Cultivated in relation to comparisons with NJAU-Wild supports a strong differentiation between cultivated and wild soybean accessions.

In the separate analysis of the EUCLEG collection the marginal likelihood of the fastSTRUCTURE-output from K = 2 to K = 10 indicated the optimum at K = 5 (Supplementary Figure 5). Although some small subgroups were formed at K > 5, we worked further with five subgroups as this clustering was in concordance with the background information of the accessions. Population structure in the EUCLEG collection is presented in Figures 1B,C and is summarized in Table 2. Additional information about the type and maturity group of accessions within each subgroup can be found in Supplementary Table 1. Of the total of 477 accessions, 362 were assigned to one of five subgroups (G1 to G5), and the remaining 115 accessions displayed substantial admixture and could not be assigned unequivocally to a specific subgroup. Some association was found between subgroup and origin namely the regions of Southern Europe, Eastern Europe, Western Europe and Northern Europe, while no clear association between subgroup and maturity group (MG) was found. Subgroup G1 contains 93 accessions, 66 of which originate from Southern Europe. They are medium late (MG 0) and late maturing (MG I/II) varieties and breeding lines from Institute of Field and Vegetable Crops, Serbia (IFVCNS), Maize Research Institute Zemun Polje, Serbia (MRIZP) and Agenzia regionale per lo Sviluppo Rurale del Friuli Venezia Giulia, Italy (ERSA FVG), and they group closely with a set of accessions from United States and Canada. Subgroup G2 contains 59 accessions, 26 of which (of all four MGs) originate from Eastern Europe; these accessions group closely with accessions from China. A total 21 of G2 accessions originate from Germany and the Czech Republic and the remaining accessions are from Bulgaria, Poland, Russia, Ukraine and China. The largest subgroup is G3 with 127 accessions, 72 of which originate from Western Europe. In G3, 57 accessions including very early (MG 000) to early maturing (MG 00) breeding lines are from Flanders Research Institute for Agriculture, Fisheries and Food, Belgium (ILVO) and Storm Seeds, Belgium. A set of 16 early maturing varieties from Canada is also part of G3. Finally, G4 and G5 contain 44 and 39 accessions, respectively. G4 contains a unique group of edamame-types from Storm Seeds (Belgium) and from Japan. Subgroup G5 contains accessions from Eastern, Western and Northern Europe; they are mainly MG 000 accessions.

**TABLE 2:** Summary of fastSTRUCTURE results for the EUCLEG collection.

Geographical Origin*	**Number of accessions**	**G1**	**G2**	**G3**	**G4**	**G5**	**Admixed**
Eastern EU	77	5	26	10	3	13	20
Southern EU	92	66	1	8			17
Western EU	179	5	13	72	30	10	49
Northern EU	14			3	1	8	2
Japan	9				7	2	
China	21		8	5		1	7
United States	11	5		3			3
Canada	33	6	1	16	2	2	6
Unknown	41	6	10	10	1	3	11
Total	477	93	59	127	44	39	115

The table shows the classification of 477 accessions in different subgroups (“G1 to G5”). “Admixed” corresponds to the group of accessions that could not be assigned unequivocally to any of the five subgroups identified by fastSTRUCTURE. For additional information about the accessions, see Supplementary Table 1.

*Bulgaria, Czech Republic, Hungary, Moldova, Poland, Romania, Russia, and Ukraine are grouped into Eastern Europe. Italy and Serbia are grouped into Southern Europe. Austria, Belgium, France, Germany, Netherlands, and Switzerland are grouped into Western Europe. Belarus, Estonia, Lithuania and Sweden are grouped into Northern Europe.

The results of the cluster analysis were similar with those of the fastSTRUCTURE analysis of EUCLEG and NJAU combined (see above). The accessions of the two collections were assigned to different clusters. NJAU-cultivated and NJAU-wild grouped into two separate clusters as in Wang et al. (2016). Interestingly, the accessions of Chinese origin included in the EUCLEG collection clustered mostly among the EUCLEG germplasm, spread over several clusters and only one of them clustered within NJAU-Cultivated (Supplementary Figure 6). This indicates that EUCLEG accessions originating from China resemble more closely with other EUCLEG accessions than the Chinese accessions from NJAU.

### Genetic Diversity in the EUCLEG and NJAU Collections

A higher number of fixed SNP sites (MAF = 0) was observed in the EUCLEG collection (20%) compared to the NJAU collections (13% and 10% in NJAU-Cultivated and NJAU-Wild, respectively) (Supplementary Figure 7). Moreover, the proportion of polymorphic SNPs (MAF ≥ 5%) was lower in EUCLEG (62%) than in NJAU-Cultivated (72%) and NJAU-Wild (82%). These results suggest an overall higher level of homozygosity in the EUCLEG collection compared to the NJAU collections.

**FIGURE 2:**
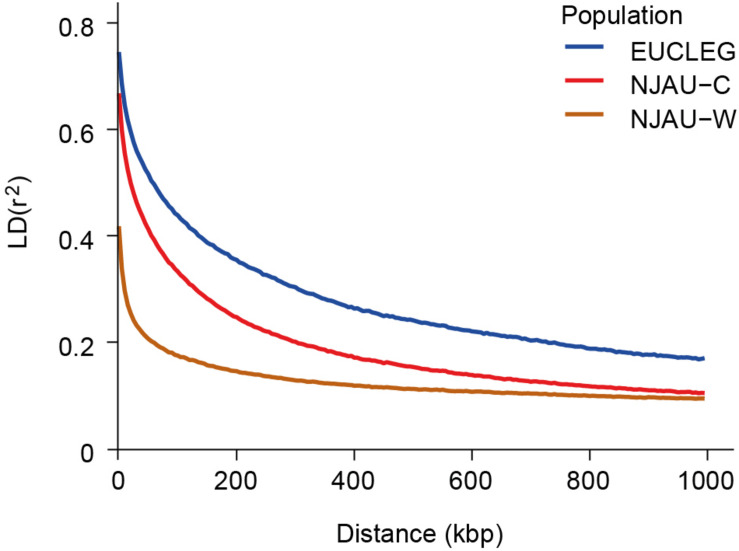
Linkage disequilibrium decay at increasing genetic distances in the EUCLEG collection, the NJAU-Cultivated collection and in the NJAU-Wild collection. X-axis and Y-axis show the average distance between two SNPs in kbp and mean linkage disequilibrium (*r*^2^) within bins of 5 kbp distance, respectively.

Linkage disequilibrium (LD) dropped to half of its maximum at 175, 100, and 50 kbp in EUCLEG, NJAU-Cultivated and NJAU-Wild, respectively (Figure 2), suggesting lower effective population size in the EUCLEG and NJAU-Cultivated collections than in the NJAU-Wild collection. In addition, the three collections showed different patterns of LD per chromosome (Table 1), which indicates different histories of recombination and selection in these three collections.

Average pairwise divergence among genotypes per site (π) decreased from 0.31 in NJAU-Wild to 0.26 in NJAU-Cultivated and to 0.23 in EUCLEG (Table 1). This is in agreement with a loss of genetic diversity due to domestication and selection in cultivated soybean. There is also a clear tendency toward less variation in average π values per chromosome in NJAU-Wild compared to NJAU-Cultivated and to EUCLEG (coefficient of variation of average π values per chromosome is 4.6, 10.4, and 13.1%, respectively). This is consistent with a scenario of a genetic bottleneck and selection that might have more prominently affected specific chromosomes in the cultivated genepools suggesting that selective sweep analysis comparing EUCLEG and NJAU-Wild may help to identify chromosomal regions that have undergone selection and domestication in the past and that probably are involved in the determination of important agronomic traits.

### Selective Sweep Analysis

**FIGURE 3:**
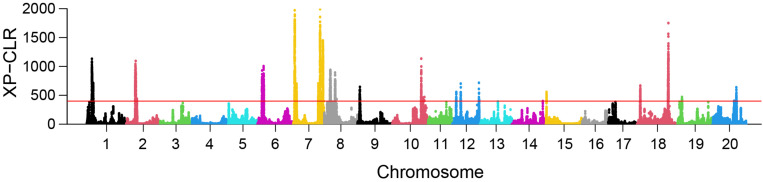
Genome-wide distribution of XP-CLR values in the comparison EUCLEG versus NJAU-Wild. The red line indicates the threshold of the 99^th^ percentile of XP-CLR values. Each dot represents the XP-CLR value obtained for a window of 1 Mbp size, sliding at steps of 5 kbp.

XP-CLR analysis revealed 23 selective sweep regions with an average length of 1.8 Mbp (range 1.14 Mbp–3.75 Mbp) (Table 3), accounting for 4% of the total sequenced genome length. Selective sweep regions were present on 12 different chromosomes including 1, 2, 6, 7, 8, 9, 10, 12, 15, 18, 19, and 20 and some chromosomes had multiple selective sweep regions (Figure 3 and Supplementary Figure 8). Exploration of SoyBase^7^ delivered 248 of the total 2,880 previously published QTLs coinciding with the selective sweep regions (Table 4 and Supplementary Table 2). A total of 3,811 genes were positioned within the selective sweep regions. The description of genes is provided in Supplementary Table 3. Consistent with the selected proportion of total chromosome size, selective sweep regions on chromosome 7 and 19 contained the highest (1,104) and the lowest (44) number of genes, respectively (Supplementary Table 3).

**TABLE 3:** Selective sweep regions determined by XP-CLR analysis between EUCLEG and NJAU-Wild.

**Selective sweep region**	**Start (bp)**	**End (bp)**	**Number of SNPs**	**Average XP-CLR**	**π**		
					**EUCLEG**	**NJAU-Cultivated**	**NJAU-Wild**
1.1	6,895,000	8,575,000	388	748	0.08	0.33	0.25
1.2	8,685,000	10,380,000	359	562	0.04	0.22	0.30
2.1	12,600,000	14,860,000	671	705	0.19	0.29	0.27
6.1	5,960,000	7,525,000	343	640	0.20	0.26	0.33
6.2	8,355,000	10,020,000	523	600	0.14	0.13	0.39
7.1	1,540,000	3,615,000	755	1,153	0.21	0.35	0.34
7.2	3,835,000	5,930,000	657	513	0.21	0.33	0.30
7.3	35,825,000	37,295,000	596	509	0.18	0.23	0.33
7.4	38,145,000	40,000,000	602	1,043	0.08	0.17	0.35
7.5	40,275,000	44,025,000	1,103	886	0.08	0.16	0.35
8.1	7,895,000	10,320,000	840	585	0.21	0.30	0.28
8.2	15,190,000	16,905,000	565	558	0.13	0.33	0.31
9.1	2,915,000	4,750,000	669	482	0.20	0.31	0.28
10.1	41,245,000	42,715,000	457	628	0.16	0.26	0.34
10.2	44,055,000	47,045,000	973	426	0.11	0.21	0.32
12.1	5,565,000	6,760,000	424	451	0.14	0.26	0.33
12.2	11,845,000	13,205,000	376	485	0.06	0.06	0.35
12.3	38,140,000	39,650,000	527	489	0.21	0.26	0.33
15.1	560,000	1,720,000	352	471	0.23	0.21	0.34
18.1	4,190,000	5,555,000	457	516	0.20	0.28	0.32
18.2	44,170,000	46,055,000	519	830	0.05	0.26	0.31
19.1	6,260,000	7,400,000	335	425	0.07	0.16	0.31
20.1	33,710,000	35,135,000	515	478	0.25	0.27	0.30
Average			565	616	0.15	0.25	0.32

*“Selective sweep region” refers to the name (left side of decimal indicates the chromosome on which the region is located and the right side is an ordinal number), “Start” and “End” delineate the chromosome coordinates of the selective sweep region. “Number of SNPs” is the total number of SNPs contained in the candidate selective sweep region. “Average XP-CLR value” is the average of XP-CLR values for all the windows contained in the selective sweep region. π is the average nucleotide diversity.*

**TABLE 4:** QTLs in the selective sweep regions.

**Selective sweep region**	**Start (bp)**	**End (bp)**	**QTL^*a*^**
1.1	6,895,000	8,575,000	FAT 9-2
1.2	8,685,000	10,380,000	PROT 7-1
2.1	12,600,000	14,860,000	AMIN 10-3, SCN 4-2, K 1-1, K 1-2, FAT 9-4, Pod 1-2, Pod 1-3, Pod 4-1
6.1	5,960,000	7,525,000	DTF 2-3, AMIN 4-1, AMIN 11-1, AMIN 12-1, AMIN 14-2, AMIN 16-1, DTF 7-3, Oil 3-5, SCN 5-12, DTF 4-22
6.2	8,355,000	10,020,000	SDS 1-5, WUE 1-29
7.1	1,540,000	3,615,000	DTF 3-5, SW 4-7, DTF 6-6, FAT 2-5, FAT 3-34, FAT 2-6, FAT 3-35, Zn 1-17, Zn 1-18, Zn 1-19, Zn 1-20, Zn 1-21, DTF 4-23, Oil 8-8, PROT 7-6
7.2	3,835,000	5,930,000	Oil 8-10, K 1-10, K 1-11, K 1-12, K 1-13, K 1-14, K 1-15, K 1-16, K 1-17, DTF 6-7, Pod 1-9, Pod 1-10, Pod 1-11, FAT 1-4, FAT 4-8, WUE 1-46
7.3	35,825,000	37,295,000	SCN 3-1, SCN 1-5, SDS 1-8, P 1-16, SDS 1-9, P 1-17, SDS 1-10, P 1-18, SDS 1-11, P 1-19, SDS 1-12, SDS 1-13, SDS 1-14, SCN 3-2, P 1-20, SDS 1-15, P 1-21, SDS 1-16, P 1-22, SCN 4-4, SDS 1-17, P 1-23, SDS 1-18, P 1-24, SDS 1-19, P 1-25, SDS 1-20, SDS 1-21, SDS 1-22, WUE 1-49, SDS 1-23, SDS 1-24, SDS 1-25, SDS 1-26, SDS 1-27, SDS 1-28, SDS 1-29, SDS 1-30, NF 1-69, NF 1-70, NF 1-71, SDS 1-31, SDS 1-32, SDS 1-33, SDS 1-34, SDS 1-35, SDS 1-36, SDS 1-37, SDS 1-38, WUE 1-50
7.4	38,145,000	40,000,000	Fe 1-8, DTF 2-9, DTF 7-9, P 1-26
7.5	40,275,000	44,025,000	SCN 5-17, SW 4-8, Mn 1-7, Oil 3-7, DTF 6-8, SCN 4-5
8.1	7,895,000	10,320,000	SC 4-1, AMIN 4-4, AMIN 18-1, AMIN 18-2, AMIN 14-3, AMIN 16-2, AMIN 20-1, AMIN 10-10, AMIN 10-11, Pod 4-11, HC 2-2, AMIN 10-12, SC 3-4, HC 2-3, SC 4-2, SC 3-5, HC 1-1, SC 1-6, HC 2-4, SCN 4-6, FAT 5-2, PROT 7-7, SC 1-7, Oil 8-13, AMIN 22-5, AMIN 26-1, AMIN 22-6, AMIN 18-3, AMIN 14-4, AMIN 20-2, AMIN 23-1, AMIN 24-1, AMIN 25-1, FAT 9-7, Salt 1-7, Mg 1-6, FAT 6-4, TH*, TV*
8.2	15,190,000	16,905,000	PUE 2-6, SIFC 1-25, SCN 3-10
9.1	2,915,000	4,750,000	DTF 8-5, Pod 1-16, Pod 1-17, Pod 1-18, AMIN 27-2, WUE 1-59, CAN 1-4, SCL 3-17, SCL 3-18, SCL 3-19, DTF 2-14, DTF 7-14, SCL 3-20, NF 1-72, SCL 3-21
10.1	41,245,000	42,715,000	K 1-28, S 1-9, B 1-13, SMV 2-6
10.2	44,055,000	47,045,000	SCN 1-9, SCN 4-7, DTM 5-3, NF 1-84, NF 1-85, CAN 1-5, DTF 5-25, DTF 5-26, DFTM 1-3, Pod 4-1, Seeds 4-5, PH 5-1, DFTM 1-4, NF 1-86, NF 1-87, NF 1-88, NF 1-89, NF 1-90, DFTM 1-5, FAT 6-7, PubDen 1-8, CAN 1-6, NF 1-91, DTF 5-27, DTF 5-28, PH 5-2, DTM 5-4, FAT 3-36, Nodes 1-2, DTF 5-29, DTF 5-30, DTM 5-5, DTF 8-6, DTM 10-7, DTF 8-2, SIFC 1-29, SCN 5-22
12.1	5,565,000	6,760,000	SW 14-3, Pod 1-24, SW 14-4, SW 3-6, SW 3-7, PubF 1-2, SW 14-5, SW 3-8, DTF 4-46, SCN 5-27
12.2	11,845,000	13,205,000	
12.3	38,140,000	39,650,000	DTM 8-11, WUE 1-6, Pod 1-25, Salt 1-9, WUE 3-24
15.1	560,000	1,720,000	DTF 4-57, DFTM 2-15, WUE 3-27, DFTM 4-15
18.1	4,190,000	5,555,000	DTF 4-69, FAT 9-9, Oil 3-10, Oil 8-23, AMIN 22-16, AMIN 10-22
18.2	44,170,000	46,055,000	P 1-34, P 1-35, P 1-36, PROT 5-2, PubF 1-3
19.1	6,260,000	7,400,000	WUE 1-94, WUE 1-95
20.1	33,710,000	35,135,000	BRA 2-1, DTF 5-64, DTF 5-65, SCN 4-16, LeafShape 1-12, LeafWidth 1-10, WUE 1-99

*“Selective sweep region” refers to the name (left side of dot indicates the chromosome on which the region is located and the right side is an ordinal number), “Start” and “End” delineate the chromosome coordinates of the selective sweep region.“QTL” is the quantitative trait locus (retrieved from SoyBase) coinciding with the respective selective sweep region.*

^*a*^QTL information was retrieved from SoyBase (Grant et al., 2010; www.soybase.org/). The original names of QTLs from SoyBase were adapted. For a more detailed description of these QTLs, see “Supplementary Table 2”. AMIN: Seed amino acid content, B: Shoot Boron, BRA: Yield component branches on main stem, CAN: Canopy cover, DFTM: Days from flowering to maturity, DTF: Days to flowering, DTM: Days to maturity, FAT: Seed fatty acid content, Fe: Shoot Fe, HC: Hilum color, K: Shoot Potassium, LeafShape: Leaflet shape, LeafWidth: Leaflet width, Mg: Shoot Mg, Mn: Shoot Mn, NF: Nitrogen fixation Ureides content, Nodes: Yield comp Number of nodes per plant, Oil: Seed oil content, P: Shoot P, PH: Plant height, Pod: Pods per plant, PROT: Seed protein, PubDen: Pubescence desnity, PubF: Pubescence form, PUE: P use efficiency, S: Shoot Sulfur, Salt: Salt tolerance, SC: Seed coat color, SCL: Sclerotinia resistance, SCN: Soybean cyst nematode, SDS: Sudden death syndrome, Seeds: Seeds per plant, SIFC: Seed isoflavone content, SMV: Soybean mosaic virus, SW: Seed weight per plant, WUE: Water use efficiency, Zn: Shoot Zinc.

* QTLs information from Kurasch et al. (2018). TH, Tofu hardiness; TV, Tofu value.

A nearly equal and relatively low nucleotide diversity (π) was observed in selective sweep regions 6.2, 12.2, and 15.1 in both EUCLEG (0.14, 0.06, and 0.23) and NJAU-Cultivated (0.13, 0.06, and 0.21) when compared to NJAU-Wild (0.39, 0.35, and 0.34) (Table 3 and Supplementary Figure 9). This indicates that the traits regulated by these regions may have undergone similar histories of selection in EUCLEG and NJAU-Cultivated. Known QTLs for water use efficiency (WUE) and time to flowering and maturity coincide with region 6.2 and 15.1 (Table 4). In addition, genes conferring resistance to pathogens or controlling time to flowering are located in these regions (Supplementary Table 3). Strikingly, no QTL coincided with selective sweep region 12.1, although this region harbors genes for important functions such as control of time to flowering (Supplementary Table 3).

Interestingly, π was extremely low (0.04 − 0.13) in selective sweep regions 1.1, 1.2, 7.4, 7.5, 8.2, 18.2 and 19.1 in EUCLEG as compared to NJAU-Cultivated (0.16 − 0.33) and NJAU-Wild (0.25 − 0.35), indicating a greater strength of selection on these regions in EUCLEG (Table 3 and Supplementary Figure 9). Such a low diversity in EUCLEG can be caused by the effects of domestication and/or selection. These regions contain known QTLs for seed fatty acids, seed oil, yield components, resistance to biotic stresses including Sclerotinia stem rot (SCL), time to flowering, and WUE (Table 4). Genes controlling flowering and maturity, resistance against pathogens, uptake of minerals, and abiotic stress response are also located in these selective sweep regions (Supplementary Table 3). Some of the candidate genes for seed isoflavone content reported by Meng et al. (2016) are located in region 8.2 (*Glyma.08G190300*, *Glyma.08G190500*).

Other selective sweep regions (2.1, 6.1, 7.1, 7.2, 7.3, 8.1, 9.1, 10.1, 10.2, 12.1, 12.3, 18.1, 20.1) have low to medium π (0.11 − 0.25) in the EUCLEG collection compared to that in NJAU-Cultivated (0.21 − 0.35) and NJAU-Wild (0.27 − 0.34) (Table 3 and Supplementary Figure 9). Of the various known QTLs coinciding with these regions, some are related to improvement traits including seed composition (protein, oil and isoflavone content), seed yield (yield components), pathogen resistance, and time to flowering and maturity (Table 4). Interestingly, some QTLs associated with tofu quality (tofu hardiness and tofu value) reported by Kurasch et al. (2018) coincide with region 8.1. Different genes known to be involved in nodulation (nodulin *MtN3*, nodulin *MtN21*; Gamas et al. (1996)), regulating Zn, Mn, Ca, and Fe uptake, involved in flowering and maturity including *E2* (*Glyma.10G221500*) and *E4* (*Glyma.20G090000*; in close proximity of selective sweep region 20.1), and genes related to hormonal control of plant growth including auxin response factor, gibberellin-regulated protein, brassinosteroid signaling, jasmonic acid biosynthesis and strigolactone biosynthesis are located in these selective sweep regions (Supplementary Table 3).

We also observed known QTLs related to hilum color and seed coat color that coincide with selective sweep region 8.1 (Table 4). This region contains a group of chalcone synthase (CHS) genes that are part of the flavonoid and anthocyanin biosynthesis pathway required for seed coat color (Akada and Dube, 1995) (Supplementary Table 3).

## Discussion

### Genotyping Non-Chinese Soybean Accessions With the 355K SoySNP Array

In soybean, different genotyping microarrays are available including the SoySNP50K iSelect Bead chip from Illumina (Song et al., 2013) and the SoySNP180K Axiom microarray from Affymetrix (Lee et al., 2015) containing probes for 52,041 and 180,961 SNPs, respectively. Here we have used the recently developed NJAU 355K Affymetrix SoySNP array containing probes for 355,595 SNPs (Wang et al., 2016). The proportion of high quality SNPs detected in this study for the EUCLEG collection corresponded quite well with that for the NJAU collection reported by Wang et al. (2016). This indicates that the 355K SoySNP microarray, which was originally developed using plant materials of Chinese origin, is also useful for genotyping soybean from non-Chinese origin. It was therefore possible to perform SNP calling on combined EUCLEG and NJAU collections. This joint analysis offered the advantage that genotyping a larger sample set minimizes the chance of misclassification of SNPs, which reduces the type I error (Mascha and Vetter, 2018). This combined SNP dataset is therefore of great value to analyze the genetic diversity available in the EUCLEG collection and to contrast this with the genetic diversity present in the Chinese collection.

### Genetic Structure of the EUCLEG and the NJAU Collections

Soybean is native to China, Japan and Korea, and has been introduced to Europe and other parts of the world (Singh and Hymowitz, 1999; Liu et al., 2017). Evidence from previous studies has shown that the earlier soybean breeding programs of different parts of the world have used Chinese soybean accessions as ancestors (Gizlice et al., 1994; Wysmierski and Vello, 2013), although an earlier study showed a clear distinction between soybean collections from United States and China (Liu et al., 2017). Similarly, population structure analysis in our study revealed a clear genetic differentiation between the EUCLEG and both the NJAU-Wild, and NJAU-Cultivated collections. This reflects breeding efforts in different parts of the world over many decades that have concentrated on improving the local adaptation of soybean to different environmental conditions. Accessions of Chinese origin included in the EUCLEG collection clustered closely with other accessions from EUCLEG instead of clustering with NJAU accessions. This is probably because these accessions have been used for breeding purposes outside China and in this way show closer relationships with their descendants.

The level of genetic diversity (π) in this study was the lowest for the EUCLEG collection, followed by NJAU-Cultivated, and with NJAU-Wild containing the highest level of genetic diversity. This agrees with the model for soybean breeding history presented by Hyten et al. (2006), in which domestication and further selection has reduced the genetic diversity in Asian germplasm. This was followed by genetic bottlenecks during introduction of soybean to other regions of the world and further selection. As a consequence, the substantially lower level of diversity in the EUCLEG collection in comparison to the NJAU collections reflects the combined effect of all three processes (domestication, introduction bottlenecks and selection).

Nevertheless, the EUCLEG collection is strongly structured, with a distribution of genetic diversity over five subgroups. In agreement with previous reports by Žulj Mihaljević et al. (2020), our analysis confirms that soybean accessions from Southern Europe are closely related to those from United States and Canada. Soybean accessions from Eastern and Western Europe contain a range of diversity as they were distributed over all five subgroups (G1 to G5). These results indicate frequent exchange of genetic resources across countries of Eastern and Western Europe, as well as the incorporation of diversity from different geographical origins including Japan, China, United States and Canada into European breeding activities (Tavaud-Pirra et al., 2009; Hahn and Würschum, 2014).

### Selective Sweeps in the EUCLEG Collection

We have applied the XP-CLR methodology to determine selective sweeps because compared to other approaches, XP-CLR is robust to determine selective sweeps even in structured populations and has a higher power to detect signals of selection. Moreover it can be used with un-phased genotyping data (Vatsiou et al., 2016). We have identified 23 selective sweep regions spread over 12 chromosomes, that together account for 4% of the total sequenced genome length. This is in accordance with Zhou et al. (2015) who similarly found 5% of the total sequenced genome length affected by selective sweeps when comparing cultivated soybean accessions from different origins to wild Chinese soybean accessions.

We have found multiple QTLs for flowering and maturity coinciding with the selective sweep regions. Both are important phenological traits relevant for adaptation of soybean to different cultivation areas. These traits are regulated by the so-called E loci (*E1* to *E10*) (Samanfar et al., 2017). Except for *E6* and *E9*, dominant alleles at other E-loci are photoperiod sensitive and confer late maturity, and photoperiod sensitivity decreases as the number of recessive alleles increases (Bonato and Vello, 1999; Destro et al., 2001; Kong et al., 2014). In this study we found two selective sweep regions in the neighborhood of loci *E2* and *E4* (region 10.2 and proximal to region 20.1, respectively), suggesting strong signals of selection at these loci in the EUCLEG collection. These results are consistent with previous reports. According to Kurasch et al. (2017) and Miladinović et al. (2018), the European soybean accessions included in their studies contained different haplotypes of four E loci (*E1* to *E4*) with the recessive *e1* and *e2* alleles being more frequently found in Central Europe and the dominant *E3* and *E4* alleles being more frequent in Southern European accessions. The genetic diversity of region 10.2 (π = 0.11) and 20.1 (π = 0.25) indicate that the *E2* locus is more fixed than the *E4* locus in the EUCLEG collection. Furthermore, the absence of any previously reported QTL coinciding with the selective sweep region 12.2 containing genes related to time to flowering and hormonal signaling (Table 3) provides the opportunity to explore this region for new QTLs. In addition, further selection efforts to increase earliness in European germplasm can either focus on the selection of recessive alleles at the *E4* locus or the exploitation of available genetic diversity present in other loci related to photoperiod sensitivity.

Surprisingly, no significant signatures of selection were detected for stem determinacy, which is an important adaptive trait affecting grain yield in soybean (Kato et al., 2019). Determinate growth habit is a domestication related trait (reviewed in Sedivy et al., 2017), as a high level of determinacy contributes to synchronous seed maturation, thus avoiding undesired variability of moisture content in the harvested material. However, determinate varieties perform less well at high latitudes (Kato et al., 2019) including a large part of Northern and Western Europe (Schori et al., 2003). We have previously shown that stem determinacy is quite variable in the EUCLEG collection (Borra-Serrano et al., 2020), which may explain why the selective sweep analysis presented here failed to obtain relevant signatures of selection for this trait.

In Europe, soybean is considered a protein crop and, together with yield, seed protein content is one of the main breeding goals (Berschneider, 2016). We have identified several signatures of selection that coincide with QTLs for these traits. In addition, selective sweep regions also contain some QTLs related to nutrient use efficiency (e.g., P, Fe, K, and Ca) indicating that they might have been selected for nutrient use efficiency. However, from the total 230 QTLs for seed protein that are described in SoyBase, only 31 were located in the selective sweep regions detected in this study. This, together with the observation that the protein content is higher in wild accessions than in cultivated accessions (Chen and Nelson, 2004) indicates that during the domestication and improvement processes, either some favorable haplotypes for high protein content might have been lost or that the diversity present at those sites might not have been exploited yet in the EUCLEG collection. Therefore, there is still room to improve seed protein content by exploiting the genetic diversity available in the EUCLEG collection for this trait.

Previously known QTLs related to high seed oil content also coincided with the selective sweep regions in the EUCLEG collection compared to NJAU-Wild. This is in line with the observation that wild soybean seeds have low oil content (Chen and Nelson, 2004). Although European soybean is not specifically bred for high oil content, soybean breeding programs in Europe have incorporated breeding materials from United States, where seed oil content is an important trait (Xavier et al., 2018). A low to medium genetic diversity of the respective selective sweep regions in the EUCLEG collection as compared to NJAU-Cultivated and NJAU-Wild suggests that these QTLs are not completely fixed in the EUCLEG collection.

Our analysis has also revealed some signatures of selection related to seed fatty acids including the polyunsaturated fatty acids (linolenic acid and linoleic acid) and monounsaturated fatty acids (oleic acid). Improving the quality of seed oil by minimizing the level of polyunsaturated fatty acids is an important consideration for improved stability of soybean oil (Clemente and Cahoon, 2009). Moreover, signatures of selection were also found for traits related to tofu quality and isoflavone content, a metabolite that helps in the prevention of chronic diseases such as cancer and cardiovascular diseases (Messina and Messina, 2010). This is relevant information for European breeding programs with a particular interest in compositional traits related to food production.

Soybean, a member of the Fabaceae family, has the ability to fix atmospheric nitrogen through symbiosis with rhizobium bacteria. It has been reported that high yielding soybean varieties have a better ability to fix nitrogen (Collino et al., 2015). While direct selection of nitrogen fixation may not have been one of the objectives of current breeding programs, we have found that some previously known QTLs related to nitrogen fixation coincide with the selective sweep regions in the EUCLEG collection. This suggests that this trait might have been improved indirectly through selection for high yield. However, from the total of 145 QTLs for nitrogen fixation reported in SoyBase 11 coincide with selective sweep regions in our analysis. This suggests that still a broad genetic diversity might be present in the EUCLEG collection which can be further used to improve nitrogen fixation.

Resistance to diseases, especially to Sclerotinia stem rot (SCL) caused by *Sclerotinia sclerotiorum*, is considered important in European soybean breeding programs because SCL is widely spread throughout Europe (Rüdelsheim and Smets, 2012). Pannecoucque et al. (2018) reported the presence of genetic variation in the level of susceptibility for SCL in 14 early maturing varieties from Europe. Sources of SCL resistance have been identified on 11 soybean chromosomes and a total of 99 QTLs have been reported in SoyBase (reviewed in Neupane et al., 2019). In our analysis, only 9 QTLs coincided with a selective sweep region. Moreover, soybean mosaic virus (SMV) can be a serious issue in Western and Northern Europe (Aper et al., 2016). Of the 18 QTLs conferring resistance to different strains of SMV reported in SoyBase, only a single QTL coincided with a selective sweep region in the EUCLEG collection. Lack of selection signals for a large number of previously reported QTLs linked to resistance to SCL and SMV suggests a high genetic diversity at the corresponding genomic loci in the EUCLEG collection. A more detailed analysis of the genetic patterns at these QTLs in the EUCLEG collection might be relevant to plan future breeding efforts to improve SCL and SMV resistance in European germplasm.

To the best of our knowledge this is one of the first studies that explores the genetic diversity of a large soybean collection relevant for breeding in Europe, in comparison to Chinese germplasm. Although we have found several selective sweeps that could be linked to useful traits in soybean through XP-CLR analysis, some methodological aspects require attention. First, genotyping using whole genome sequencing rather than microarray data could provide more variants and thus a more detailed description of the genomic regions that have experienced selective sweeps (Ronen et al., 2013). For example, this would enable a gene enrichment analysis which ultimately could provide information about candidate genes. Second, some of the selective sweeps determined by XP-CLR might be false positives caused by demographic processes such as bottlenecks or population expansions (Weigand and Leese, 2018). While the first aspect can be tackled if sufficient resources are available, d it is hard to entirely overcome the second limitation unless other methods are developed that allow to differentiate among the different scenarios that can lead to a positive signal of selection.

## Conclusion

The present study focused on the exploration of a representative sample of soybean accessions relevant for breeding in Europe, the EUCLEG collection. This is one of the first studies in which the patterns of genetic diversity in a large soybean germplasm set relevant for breeding in Europe has been compared to the genetic diversity contained in Chinese cultivated and wild soybean germplasm. Our study has demonstrated a relatively lower genetic diversity in the EUCLEG collection compared to Chinese collections of cultivated and wild accessions, which indicates a narrow genetic base of the EUCLEG collection. However, a more detailed analysis of the patterns of genetic diversity in the EUCLEG collection has revealed substantial sub-structuration in five subgroups associated with geographical origins, and without a clear association with maturity classes. A selective sweep analysis has revealed the presence of multiple signatures of selection in the EUCLEG collection, compared to Chinese wild germplasm. In particular, genomic regions previously reported to influence grain protein, yield and disease resistance have been identified, whose exploration in future work might facilitate further selection efforts. No signals of selection have been detected for loci involved in stem determinacy, probably because no directed selection has been performed for this trait among the germplasm represented in the EUCLEG collection. On the other hand, clear signatures of selection were detected for at least two loci involved in photoperiod sensitivity and time to flowering, which are main traits considered by breeders in order to adapt soybean for cultivation in Europe (only maturity classes 000 to I/II). Taken together, our results have identified relevant genomic regions that can be further exploited to improve soybean for the European agricultural sector, either through further improvement of genetic resources that are available in Europe, or through incorporation of exotic soybean material in European breeding programs.

## Data Availability Statement

The original contributions presented in the study are included in the article/Supplementary Material, further inquiries can be directed to the corresponding author/s.

## Author Contributions

AS, HM, JA, and IR-R conceived the study. AS and HM performed the research and analyzed the data. AS and IR-R drafted the manuscript. AS, HM, JA, TR, JW, DY, and IR-R interpreted the results and elaborated the manuscript. All authors read and approved the final version of the manuscript.

## Conflict of Interest

The authors declare that the research was conducted in the absence of any commercial or financial relationships that could be construed as a potential conflict of interest. The reviewer, MS, declared a past co-authorship with one of the authors, TR, to the handling editor.

## Footnotes

1 http://www.eucleg.eu/

2 https://www.qiagen.com/be/resources/

3 https://www.thermofisher.com/

4 https://www.soybase.org/GWAS/list.php

5 https://data.jgi.doe.gov/refine-download/phytozome2

6 https://www.uniprot.org/

7 https://www.soybase.org/GWAS/list.php

## Supplementary Material

Supplementary Figure 1 Distribution of SNP categories. PHR, PolyHighResolution; MHR, MonoHighResolution; NMH, NoMinorHomozygote; OTH, Other; OTV, Off-TargetVariants; CRT; CallRateBelowThreshold. EUCLEG, NJAU and Combined represents EUCLEG separate analysis, NJAU separate analysis according to Wang et al. (2016), and EUCLEG and NJAU combined analysis, respectively. PHR, MHR, and NMH are considered recommended categories; OTH, OTV and CRT are considered non-recommended categories. The SNP categories refer to the classification made according to the Axiom^TM^ Analysis Suite 3.1 user guide (www.thermofisher.com/). Y-axis shows the frequency of SNPs (total 335,595).

Supplementary Figure 2 Frequency distribution of 224,973 SNPs from 355K SoySNP with a certain spacing distance.

Supplementary Figure 3 Results of the fastSTRUCTURE analysis of EUCLEG and NJAU combined. K (X-axis) represents the number of clusters considered. The Y-axis represents the log likelihood of the model for different number of clusters.

Supplementary Figure 4 Principal Components analysis (PCA) of the EUCLEG and NJAU combined collection. Blue, red, and orange colors represent the EUCLEG, NJAU-Cultivated and NJAU-Wild collections respectively. PC1 and PC2 explained 44 and 22% of total genotypic variation in the EUCLEG and NJAU combined collection, respectively.

Supplementary Figure 5 Results of the fastSTRUCTURE analysis of the EUCLEG collection. K (X-axis) represents the number of clusters considered. The Y-axis represents the log likelihood of the model for different number of clusters.

Supplementary Figure 6 Dendrogram representing the genetic relationships within EUCLEG and NJAU combined. “G1” to “G5” are the five subgroups identified by fastSTRUCTURE. “Admixed” are the accessions that could not be assigned unequivocally to one of the subgroups (see main text for further details). “NJAU-C” and “NJAU-W” represent the NJAU-Cultivated and NJAU-Wild parts of NJAU collection. “China” corresponds to the accessions of Chinese origin that were included in the EUCLEG collection (a total of 21 accessions, indicated by a black bar in front of the tree branch ends).

Supplementary Figure 7 Polymorphism inEUCLEG and NJAU. NJAU-C, and NJAU-W represents the cultivated and wild subpopulations of NJAU respectively. Y-axis shows the frequency of recommended SNPs (total 224,993).

Supplementary Figure 8 Results of the XP-CLR analysis comparing EUCLEG and NJAU-Wild. X-axis represents the physical position and Y-axis represents the XP-CLR value. The red line represented in each plot corresponds to the 99th percentile of the genome-wide XP-CLR values.

Supplementary Figure 9 Comparison of the genetic diversity (π) between EUCLEG (blue), NJAU-Cultivated (red) and NJAU-Wild (orange). The lines represent the average π value of the 1 Mbp windows considered in the XP-CLR analysis. X-axis and Y-axis are represent the genomic position in million base pairs (Mbp) and π value for each chromosome, respectively. The shaded areas represent the selective sweep regions revealed by XP-CLR analysis. The start and end positions of 1 Mbp window with a step size of 5 kbp were defined externally from bedtools (v2.29.2) because XP-CLR does not provide that information in the output. XP-CLR considers recombination frequency instead of actual physical positions to define a window. This is the reason why some shaded areas are shifted a little at both sides of the actual window from XP-CLR (i.e., chromosome 19 and 20) in the representation.
