# Supplementary material for: A Genome-Wide Genetic Diversity Scan Reveals Multiple Signatures of Selection in a European Soybean Collection Compared to Chinese Collections of Wild and Cultivated Soybean Accessions
Source: Front Plant Sci. 2021 Feb 26;12:631767. doi: 10.3389/fpls.2021.631767 (PMC7959735; doi:10.3389/fpls.2021.631767)
Supplement: Supplementary Figure 1 — Distribution of SNP categories. PHR, PolyHighResolution; MHR, MonoHighResolution; NMH, NoMinorHomozygote; OTH, Other; OTV, Off-TargetVariants; CRT; CallRateBelowThreshold. EUCLEG, NJAU and Combined represents EUCLEG separate analysis, NJAU separate analysis according to Wang et al. (2016), and EUCLEG and NJAU combined analysis, respectively. PHR, MHR, and NMH are considered recommended categories; OTH, OTV and CRT are considered non-recommended categories. The SNP categories refer to the classification made according to the AxiomTM Analysis Suite 3.1 user guide (www.thermofisher.com/). Y-axis shows the frequency of SNPs (total 335,595). [file Data_Sheet_1.docx]

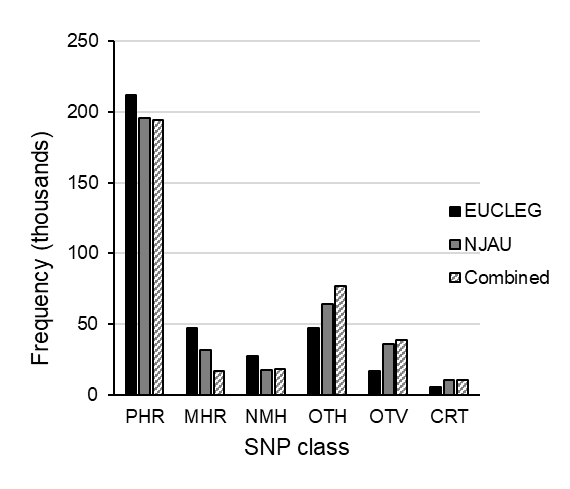


**Supplementary Figure 1.** Distribution of SNP categories. PHR; PolyHighResolution, MHR; MonoHighResolution, NMH; NoMinorHomozygote, OTH; Other, OTV; Off-TargetVariants; CRT; CallRateBelowThreshold. EUCLEG, NJAU and Combined represents EUCLEG separate analysis, NJAU separate analysis according to Wang et al. (2016), and EUCLEG and NJAU combined analysis, respectively. PHR, MHR and NMH are considered recommended categories; OTH, OTV and CRT are considered non-recommended categories. The SNP categories refer to the classification made according to the Axiom™ Analysis Suite 3.1 user guide^[[1]](#footnote-1)^. Y-axis shows the frequency of SNPs (total 335,595).


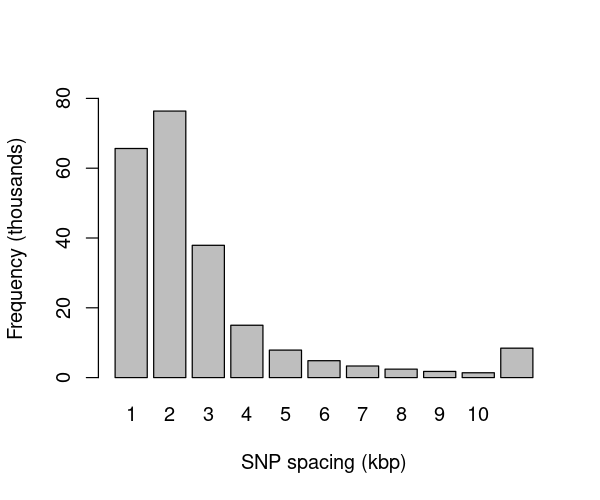


**Supplementary Figure 2.** Frequency distribution of 224,973 SNPs from 355K SoySNP with a certain spacing distance.


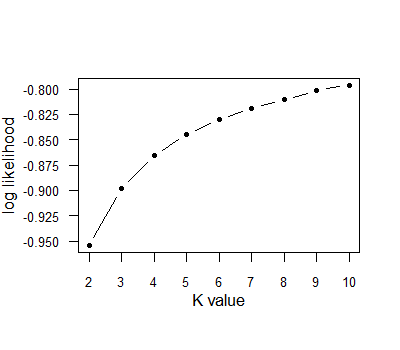


**Supplementary Figure 3.** Results of the fastSTRUCTURE analysis of EUCLEG and NJAU combined. K (X-axis) represents the number of clusters considered. The Y-axis represents the log likelihood of the model for different number of clusters.


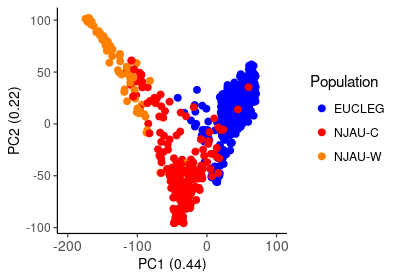


**Supplementary Figure 4.** Principal Components analysis (PCA) of the EUCLEG and NJAU combined collection. Blue, red and orange colors represent the EUCLEG, NJAU-Cultivated and NJAU-Wild collections respectively. PC1 and PC2 explained 44% and 22% of total genotypic variation in the EUCLEG and NJAU combined collection, respectively.


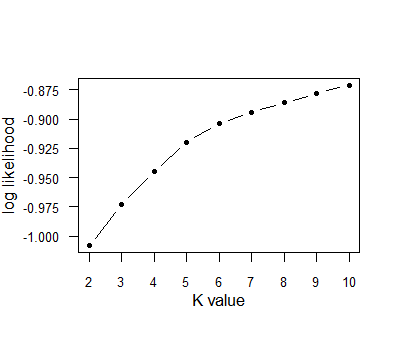


**Supplementary Figure 5.** Results of the fastSTRUCTURE analysis of the EUCLEG collection. K (X-axis) represents the number of clusters considered. The Y-axis represents the log likelihood of the model for different number of clusters.

**
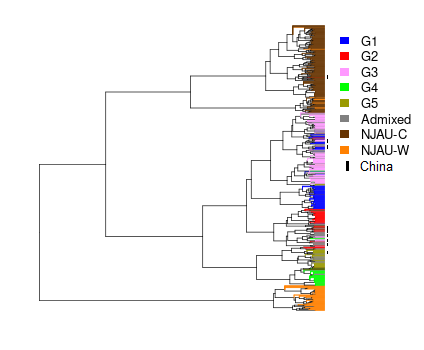
**

**Supplementary Figure 6.** Dendrogram representing the genetic relationships within EUCLEG and NJAU combined. ‘G1’ to ‘G5’ are the five subgroups identified by fastSTRUCTURE. ‘Admixed’ are the accessions that could not be assigned unequivocally to one of the subgroups (see main text for further details). ‘NJAU-C’ and ‘NJAU-W’ represent the NJAU-Cultivated and NJAU-Wild parts of NJAU collection. ‘China’ corresponds to the accessions of Chinese origin that were included in the EUCLEG collection (a total of 21 accessions, indicated by a black bar in front of the tree branch ends).


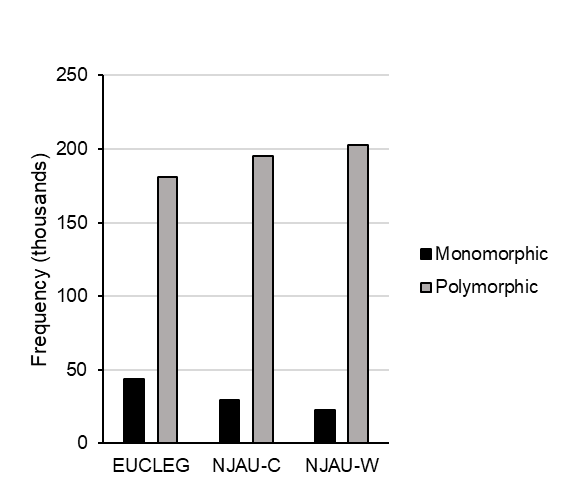


**Supplementary Figure 7.** Polymorphism in EUCLEG and NJAU collections. NJAU-C, and NJAU-W represents the cultivated and wild subpopulations of NJAU respectively. Y-axis shows the frequency of recommended SNPs (total 224,993).

| 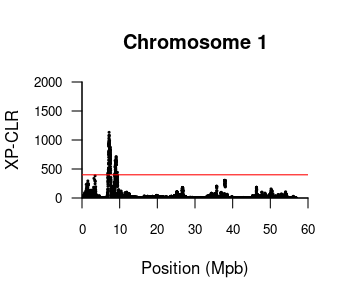 | 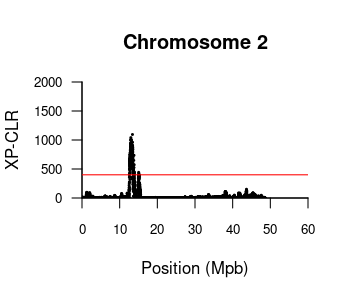 | 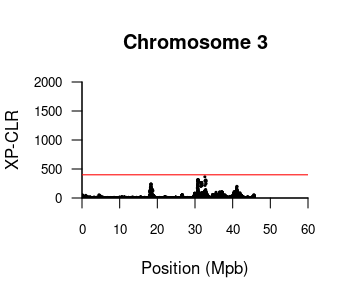 |
| --- | --- | --- |
| 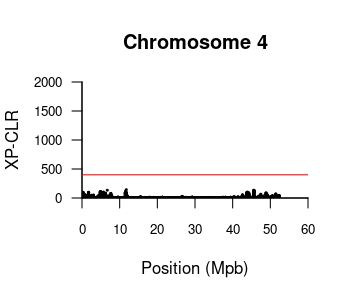 | 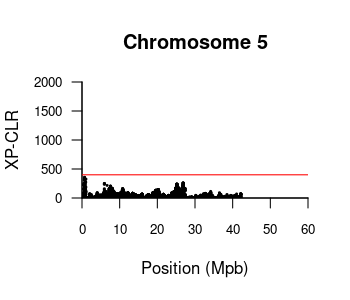 | 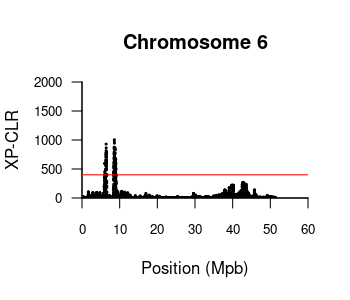 |
| 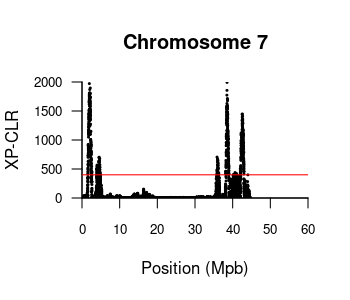 | 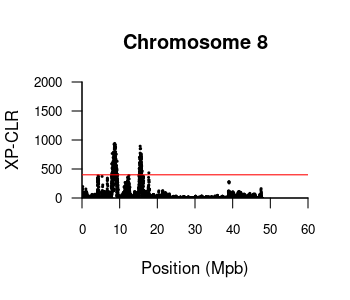 | 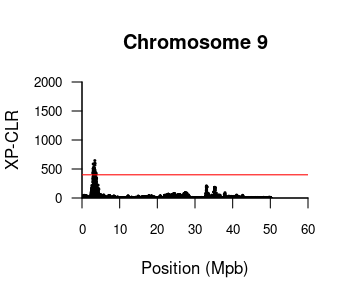 |
| 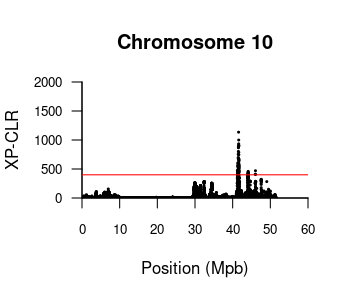 | 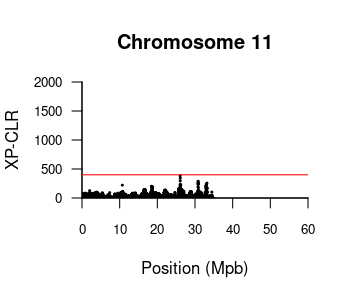 | 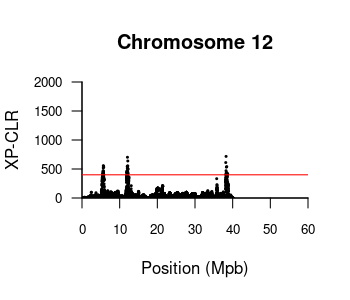 |
| 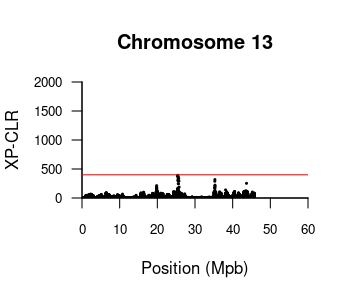 | 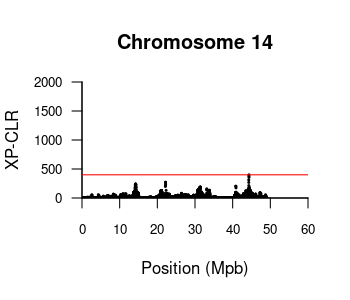 | 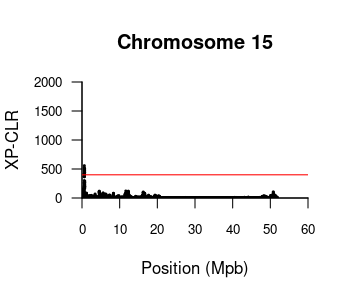 |
| 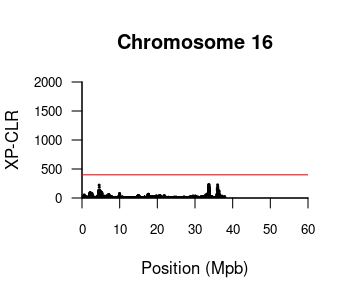 | 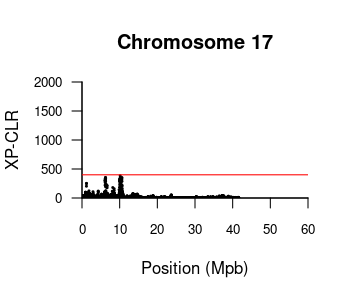 | 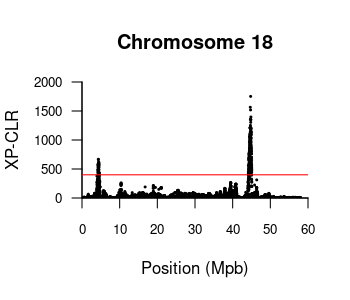 |
| 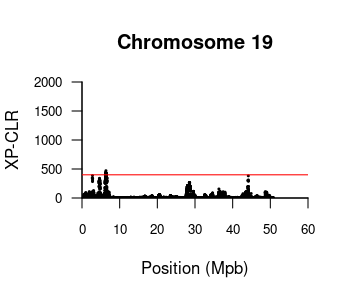 | 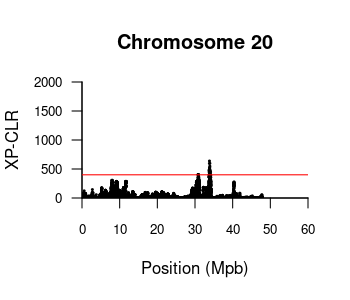 |  |

**Supplementary Figure 8.** Results of the XP-CLR analysis comparing EUCLEG and NJAU-Wild. X-axis represents the physical position and Y-axis represents the XP-CLR value. The red line represented in each plot corresponds to the 99th percentile of the genome-wide XP-CLR values.

| 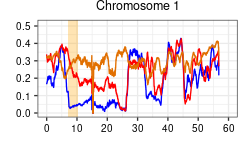 | 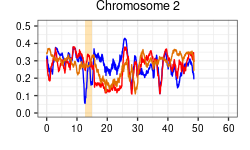 | 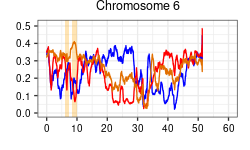 |
| --- | --- | --- |
| 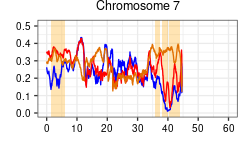 | 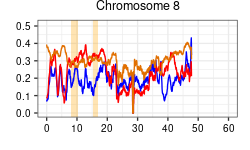 | 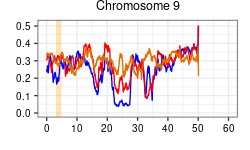 |
| 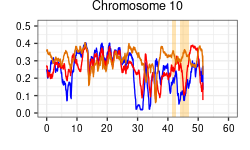 | 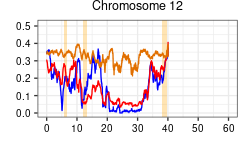 | 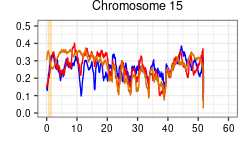 |
| 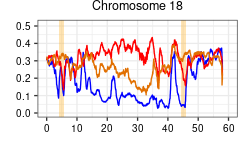 | 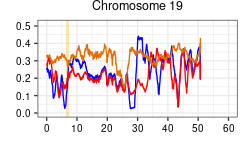 | 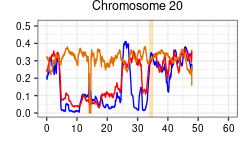 |

**Supplementary Figure 9.** Comparison of the genetic diversity (π) between EUCLEG (blue), NJAU-Cultivated (red) and NJAU-Wild (orange). The lines represent the average π value of the 1 Mbp windows considered in the XP-CLR analysis. X-axis and Y-axis are represent the genomic position in million base pairs (Mbp) and π value for each chromosome, respectively. The shaded areas represent the selective sweep regions revealed by XP-CLR analysis. The start and end positions of 1 Mbp window with a step size of 5 kbp were defined externally from bedtools (v2.29.2) because XP-CLR does not provide that information in the output. XP-CLR considers recombination frequency instead of actual physical positions to define a window. This is the reason why some shaded areas are shifted a little at both sides of the actual window from XP-CLR (i.e. chromosome 19 and 20) in the representation.

1. <https://www.thermofisher.com/> [↑](#footnote-ref-1)
